# Disparities in the use of antenatal care service in Ethiopia over a period of fifteen years

**DOI:** 10.1186/1471-2393-13-131

**Published:** 2013-06-15

**Authors:** Elias Ali Yesuf, Ronit Calderon-Margalit

**Affiliations:** 1 Department of Health Services Management, Jimma University, Jimma, Ethiopia; 2 Braun School of Public Health and Community Medicine, Hadassah-Hebrew University, Jerusalem, Israel

**Keywords:** Inequities, Residence, Region, Economic status, Education, Antenatal care, Ethiopia

## Abstract

**Background:**

Little is known about factors contributing to inequities in antenatal care use in Ethiopia. We aimed to assess inequities in the use of antenatal care on the basis of area of residence, administrative region, economic status and education.

**Methods:**

This study was based on data from repeated cross-sectional surveys carried out by Measure Demographic and Health Survey and Central Statistical Authority of Ethiopia. The surveys were conducted in February-June 2000, April-August 2005, and December 2010-June 2011. The surveys employed a cluster sampling design to select a nationally representative sample of 15–49 year-old women. The main outcome variable was at least one antenatal care visit for the last live birth in the 5 years preceding the surveys. Statistical analysis was completed by applying the sampling weights in order to consider the complex sampling design.

**Results:**

A total of 7978, 7307 and 7908 weighted number of women participated in the three surveys, respectively. The rate of antenatal care coverage in Ethiopia has increased from 26.8% in 2000 to 42.7% in 2011. The odds of antenatal care use were 2.4 (95% CI: 1.7-3.2, p < 0.0001), 1.6 (95% CI: 1.2-2.2, p = 0.003) and 1.8 (95% CI: 1.3-2.6, p = 0.001) times higher among women from urban areas than those from rural areas at the three time points, respectively. The odds ratio of antenatal care use among women with secondary or higher education compared with women of no education increased from 2.6 (95% CI: 2.0-3.4, p < 0.0001) in 2000 to 5.1 (95% CI: 2.8-9.4, p < 0.0001) in 2011. Moreover, the odds of use among women from the richest households at the three time points were 2.7 (95% CI: 2.1-3.6, p < 0.0001), 4.4 (95% CI: 3.3-6.0, p < 0.0001), and 3.9 (95% CI: 2.8-5.5, p < 0.0001) times higher compared with their counterparts from the poorest households. Furthermore, we have observed a wide regional variation in the use of ANC in Ethiopia.

**Conclusions:**

The wide inequities between urban and rural areas, across economic and educational strata in the use of antenatal care highlight the need to put more resources to poor households, rural areas, and disadvantage regions. We suggest further study to understand additional factors for the deep unmet need in rural areas and some regions of Ethiopia.

## Background

The Maternal Mortality Ratio (MMR) in Ethiopia has declined by only 22% in the last decade from 871 per 100 000 live births in the year 2000 [1] to 676 per 100 000 live births in 2011 [2]. In order to achieve the 5th Millennium Development Goal (MDG) – of reducing maternal mortality by 75% in the year 2015 from the 1990 baseline level- access to adequate and high quality maternal health services including basic antenatal care (ANC), intrapartum care and postnatal care is essential [3].

ANC provides an opportunity for skilled providers so that they may identify and treat or refer complications [3]. Moreover, it was reported that in sub-Saharan Africa, women having four ANC visits are 7.3 times more likely to give birth in a health facility than those with no antenatal visits [4]. In Bolivia, the odds of modern contraceptive use of women with at least one ANC visit during pregnancy were about 4.3 times than those without [5]. A combination of a 60% prevalence of contraceptive use, improved transport and birth in a health facility can reduce 75% of the maternal deaths [6].

However, studies have documented that the ANC services are not equally distributed across the different socioeconomic groups. According to Barros and colleagues, in 54 of the 75 countries investigated in the Countdown to 2015 study, the coverage of antenatal care with a skilled provider was 77.9% (95% CI: 71.4-93.2), the rate ratio of use among the richest women relative to the poorest being 1.9 (95% CI: 1.2-1.9) [7].

Differences in antenatal care use were attributed to constraints in the supply side related to physical access, user fees and quality of the services. In a systematic review by Say and Raine, the reorganization of the health care system in Morocco has introduced fees for maternal health services that may have limited access to poor women [8].

Constraints in demand are equally important in determining the use of maternal health services, and may operate at the individual, household and community level. At the individual level, age, income, education and knowledge about the characteristics of, and need for health services may limit women from seeking maternal health care. In addition, culturally, women may have no autonomy to seek care when husbands or in-laws are conceived as the decision makers on care seeking practices for women [9].

As a strategy for tackling the supply side constraints -starting from 1996/1997- Ethiopia has started an ongoing ambitious national program, the Health Sector Development Program (HSDP). Key objectives of this program are universal coverage of primary health care and reduction of maternal mortality from 871 to 600 per 100 000 live births [10]. It is important to address disparities if the country is to achieve these targets.

So far, few studies have examined the determinants of ANC use in Ethiopia. In rural south Ethiopia, ANC use by women was inversely associated with age. In that study women aged 25–34 years were 43% less likely to use ANC compared to younger women [11]. Another study demonstrated lack of association between autonomy level in Ethiopian women, reflected by women being the sole decision-makers during daily household purchases, and use of ANC care [12]. According to a nationally representative study conducted in the 2000, urban residence and secondary/ higher education were found to be independent predictors of at least 1 ANC visit with odds ratios (OR) of 4.1 (3.1-5.5) and 4.0 (2.7-5.9), respectively [13]. This study did not control for economic status and work autonomy.

These studies were somewhat limited in scope involving single communities, focused on only one aspect of the inequity (women’s age or autonomy), or were not based on recent data with the control of major potential confounders. Therefore, an additional study of the factors contributing to inequity in the use of ANC service in Ethiopia was indicated. Furthermore, trends in an inequity in the use of ANC need to be investigated in order to inform policy debate.

The aim of this study was to better understand the disparities in ANC use across the urban/rural divide, the socioeconomic groups, the educational strata and regions of Ethiopia and the corresponding trends in inequity over time. Ultimately, this information will be used to target national and regional programs to the disadvantaged and marginalized groups of the Ethiopian society.

## Methods

This study was based on nationally representative repeated cross-sectional surveys conducted by the Measure Demographic and Health Surveys (DHS) Corporation of the United States of America (USA) and the Central Statistical Authority (CSA) of Ethiopia in 2000, 2005, and 2011. These community-based surveys encompassed all administrative regions of Ethiopia, and included both urban and rural areas [1,2,14].

The current analysis included women of reproductive age (15–49 years) who have had at least one live birth in the five years prior to the time of interview, and who participated in one of the three surveys.

A two-stage stratified-cluster sampling was used for all the three surveys. In both the 2000 and the 2005 Ethiopia DHS, the sampling was based on the Census Enumeration Areas (CEAs) of the 1994 Population and Housing Census of Ethiopia [15]. First, a sample of 540 (139 urban and 401 rural) CEAs was selected using systematic sampling from all regions. Then, 27 and 24–32 households per CEA were selected systematically in 2000 and 2005, respectively. This resulted in a total of 14 642 households and 15 716 eligible women in 2000, and 14 645 households and 14 717 eligible women in 2005. The 2011 survey was based on the 2007 National Population and Housing Census of Ethiopia [16]. This survey has sought to select 624 CEAs (187 urban and 437 rural) resulting in 18 720 households and 17 385 eligible women. Response rates for the first, second, and third surveys were 97.8% (n = 15 370), 95.6% (n = 14069), and 95% (n = 16 515), respectively.

In-person interviews were conducted by trained interviewers using structured questionnaires. As mentioned above, women were eligible for this study if they had at least 1 live birth in the five years preceding the survey. If a woman had more than 1 live birth in that period, she was asked about the most recent live birth (index pregnancy).

### Study outcome

The use of ANC (yes versus no) was defined as at least one visit to a doctor; nurse; midwife; or trained traditional birth attendant (for the 2000 survey), or health extension worker (for the 2005 and 2011 surveys) for the supervision of the last pregnancy which has resulted in a live birth as stated by the respondent.

### Study predictors

Residence (urban versus rural) was defined according to the CEA classification. Ethiopia is subdivided into regions which are semi-autonomous administrative entities with geographic boundaries and specific ethnic distribution. For the purpose of this study, Afar, Somali, and Harari administrative regions were collapsed into a single category because they are predominantly nomadic and share similar customs and traditions, with a solid majority of the population being Muslim. In addition, Gambella and Benshangul-Gumuz regions were grouped together as they share the same geographic area. Finally, Addis Ababa and Dire Dawa administrative regions were categorized as a single entity, because both regions are almost entirely urban.

In order to measure economic status, we constructed a composite variable called household economic status index as recommended by Filmer and Pritchett [17]. The economic status index is based on household utilities and assets. It includes: utilities (source of drinking water and electricity), type of toilet facility, main floor material of the house, main roof material of the house, type of cooking fuel used by the household, and media assets (radio and television). All variables were used as dichotomous variables and were included in a Principal Components Analysis (PCA), to generate weights for each component of the economic status index. A weighted economic score was generated and was divided into quintiles, presented as the five economic status levels (poorest, poorer, middle, richer and richest). This method produced similar results to the ones provided by the Measure DHS and CSA of Ethiopia for the 2005 and 2011 surveys. Therefore, for these surveys, we have used the wealth quintiles provided by Measure DHS and CSA of Ethiopia; the 2000 survey did not have in-built wealth quintiles from the outset, therefore, we used our calculated ones.

Education was defined as the highest level of schooling attained and was measured on an ordinal scale (no education, primary education and secondary or higher education).

Work status of women is an indicator of employment and participation in a paid labor [18]. It was employed as an indirect measure of one dimension of woman’s autonomy in household decisions, i.e., work autonomy. A composite variable was constructed comprising the sum of work status (not working = 0, working = 1), employer (someone else = 1, family member = 2, self employment = 3), place of work (home = 1, away = 2), employment condition (seasonal or occasional = 1, permanent = 2), and earning type (payment in kind = 1, mixed = 2, cash only = 3). A score of zero was categorized as no autonomy. In the 2000 survey, the median score for the work autonomy was 7; whereas in the 2005 and the 2011 surveys, it was 6. At or below median score on the work autonomy was considered as low autonomy and above median score was considered as high autonomy.

Attitude towards wife beating (positive versus negative) was formulated as a composite variable. A woman was regarded as having a positive attitude towards spousal violence if she stated that she should be beaten in at least one of the following conditions: neglecting child, arguing with her spouse, refusing sex, going out without the permission of her spouse or burning food.

Additional variables included age at the time of interview (15–24, 25–34 and 35+ years), parity (1, 2–4, and 5+ children), and marital status (married versus others).

For all statistical procedures, analyses were restricted to individuals with complete information on the variables required for a particular analysis. The maximum percentage of missing values was 6%.

### Statistical methods

Characteristics of the respondents were described using frequencies and percentages. Univariate analysis was used to determine the association of individual independent variables with the dependent variables, the results of which are presented as unadjusted odds ratios (ORs) with 95% confidence intervals (CIs). These analyses were conducted using IBM Statistical Package for Social Sciences (SPSS, Chicago, Il.) version 19.0.

Multivariable logistic regression models were used to control for potential confounding variables, taking into account the sampling weights, using STATA 12.0 (Statacorp, Tx.). The results of these models are presented as adjusted ORs (AOR) with 95% CIs. Both for bivariate and multivariate models, the observed level of statistical significance were set at 5%. The multivariable models were checked for evidence of lack of goodness of fit as suggested by Hosmer and Lemeshow [19]. A p-value of < 0.3 was considered as an evidence of the lack of goodness of fit.

### Ethical approval

Ethical approval for the surveys was provided by the Institutional Review Boards of Science and Technology Commission (later Ministry of Science and Technology) of Ethiopia and Measure DHS, Calverton, Maryland, USA. Moreover, the respondents provided written consent to participate in the surveys.

## Results

### Demographic characteristics of the participants

In the 2000 survey, a total of 7978 weighted number of women of child bearing age (mean age: 29.7 ± 7.5 years) had participated. Of those, 88.6% were from rural areas, and nearly 88% were from three administrative regions, namely, Oromia, Amhara and SNNPR. About 82% of participants had no education. About half of the participants were Ethiopian Orthodox Christians, 90% were married, and 42% were grand multipara (had a parity of ≥5). As shown in Table 1, the demographic characteristics of the two subsequent surveys were similar to that of the year 2000 (Table 1).

**Table 1:** Socio-demographic characteristics of antenatal care survey participants, Ethiopia

**Characteristics**	**2000**				**2005**				**2011**			
	**Weighted**		**Unweighted**		**Weighted**		**Unweighted**		**Weighted**		**Unweighted**	
	**Number**	**%**	**Number**	**%**	**Number**	**%**	**Number**	**%**	**Number**	**%**	**Number**	**%**
**Residence**												
Urban	908	11.4	1286	17.8	634	8.7	1054	16.0	1188	15.0	1513	19.5
Rural	7070	88.6	5959	82.2	6674	91.3	5535	84.0	6720	85.0	6251	80.5
**Region**												
Tigray	536	6.7	751	10.4	480	6.6	671	10.2	530	6.7	847	10.9
Amhara	2224	27.9	1128	15.6	1856	25.4	1032	15.7	1991	25.2	965	12.4
Oromiya	3059	38.3	1343	18.5	2723	37.3	1211	18.4	3116	39.4	1100	14.2
SNNPR	1695	21.2	1037	14.3	1632	22.3	1129	17.1	1634	20.7	1053	13.6
Afar,Harari,Somali	186	2.3	1210	16.7	372	5.1	1112	16.9	295	3.7	1713	22.1
Ben-Gumuz,Gambela	103	1.3	991	13.7	92	1.3	845	12.8	123	1.6	1282	16.5
Addis Ababa,Dire Dawa	175	2.2	785	10.8	153	2.1	589	8.9	219	2.8	804	10.4
**Education**												
No education	6550	82.1	5732	79.1	5734	78.5	4956	75.2	5270	66.6	5184	66.8
Primary education	1003	12.6	933	12.9	1205	16.5	1060	16.1	2270	28.7	2095	27.0
Secondary and higher	425	5.3	580	8.0	368	5.0	573	8.7	368	4.7	485	6.2
**Age**												
15-19	472	5.9	434	6.0	440	6.0	437	6.6	402	5.1	416	5.4
20-24	1727	21.6	1516	20.9	1473	20.2	1384	21.0	1608	20.3	1596	20.6
25-29	2028	25.4	1936	26.7	1961	26.8	1810	27.5	2383	30.1	2292	29.5
30-34	1496	18.7	1413	19.5	1428	19.5	1258	19.1	1489	18.8	1507	19.4
35-39	1219	15.3	1125	15.5	1138	15.6	1000	15.2	1239	15.7	1203	15.5
40-44	706	8.9	576	8.0	578	7.9	478	7.3	571	7.2	550	7.1
45-49	330	4.1	245	3.4	290	4.0	222	3.4	216	2.7	200	2.6
**Religion**												
Orthodox	4057	50.9	3234	44.6	3262	44.6	2774	42.1	3327	42.1	2694	34.7
Protestant	1232	15.4	997	13.8	1404	19.2	1159	17.6	1763	22.3	1479	19.0
Moslem	2337	29.3	2719	37.5	2382	32.6	2425	36.8	2563	32.4	3359	43.3
Others*	352	4.4	295	4.1	259	3.5	231	3.5	250	3.2	228	2.9
Missing	-	-	-	-	-	-	-	-	5	0.1	4	0.1
**Marital status**												
Married	7193	90.2	6492	89.6	6772	92.7	6058	91.9	6765	85.5	6624	85.3
Others	785	9.8	753	10.4	535	7.3	531	8.1	1143	14.5	1140	14.7
**Birth Order**												
1	1362	17.1	1391	19.2	1190	16.3	1232	18.7	1399	17.7	1477	19.0
2-4	3264	40.9	3002	41.4	3026	41.4	2830	43.0	3464	43.8	3392	43.7
5+	3352	42.0	2852	39.4	3092	42.3	2527	38.4	3045	38.5	2895	37.3
**Total**	**7978**	**100.0**	**7245**	**100.0**	**7307**	**100.0**	**6589**	**100.0**	**7908**	**100.0**	**7764**	**100.0**

*Catholic, Traditional and other unspecified.

### ANC visits in Ethiopia

Table 2 presents weighted rates per 100 participants and crude OR for at least one ANC visit, by the study covariates. In 2000, 2138 out of 7978 (26.8%) mothers attended at least 1 ANC in their last pregnancy, and only 10.4% (n = 830) had the recommended 4 or more ANC visits. In 2005, 2053 out of 7307 (28.1%) weighted number of women had at least 1 ANC visit, and 12.2% (n = 891) had the recommended 4 or more visits. A substantial increase was shown in 2011, where 42.5% (3365/7908) of women had at least 1 ANC visit, and 19.1% (n = 1508) had the recommended 4 or more visits. In all three surveys, the lowest rate of ANC visits was found in women aged 35 years or more (Table 2). No large differences were noted by either religion or marital status; however, the use of ANC had consistently decreased with increasing birth order.

**Table 2:** **Weighted percentage points and unadjusted odds ratios (OR) and 95**% **confidence interval (CI) for at least 1 antenatal care visit by sociodemographic characteristics of the participants and survey year, Ethiopia**

**Characteristics**	**2000**			**2005**			**2011**		
	**Number**	**%**	**Crude OR (95% CI)**	**Number**	**%**	**Crude OR (95% CI)**	**Number**	**%**	**Crude OR (95% CI)**
**Residence**	**7928**			**7277**			**7880**		
Urban	890	67.2	7.3(6.3,8.5)^c^	630	69.7	7.2(6.0,8.6)^c^	1167	76.5	5.6(4.8,6.5)^c^
Rural	7038	21.9	1	6647	24.3	1	6713	36.8	1
**Region**	**7928**			**7276**			**7883**		
Tigray	536	36.9	2.2(1.8,2.7)^c^	479	37.4	1.5(1.2,1.9)^c^	529	64.5	2.7(2.2,3.3) ^c^
Amhara	2219	18.8	1	1851	26.7	1	1972	40.3	1
Oromiya	3044	27.7	1.6(1.3,2.0) ^c^	2713	25.2	0.9(0.8,1.1)	3116	39.5	1.0(0.9,1.1)
SNNPR	1674	28.3	1.8(1.5,2.2) ^c^	1624	31.2	1.3(1.1,1.6)^b^	1631	40.5	1.0(0.9,1.2)
Afar, Harari, Somali	183	23.5	1.7(1.4,2.0) ^c^	369	10.3	0.7(0.5,0.8)^c^	295	30.2	0.6(0.5,0.8)^b^
Ben-Gumuz, Gambela	103	31.1	2.2(1.8,2.7) ^c^	90	27.8	1.3(1.1,1.6)^b^	123	44.7	1.2(0.8,1.7)
Addis Ababa, Dire Dawa	169	78.7	10.1(8.1,12.5) ^c^	150	82.7	7.3(5.8,9.1)^c^	217	90.3	13.7(8.7,21.7)^c^
**Economic status**	**7502**			**7276**			**7881**		
Poorest	1577	17.7	1	1518	13.5	1	1737	25.1	1
Poorer	1932	19.8	1.2(1.0,1.4)	1547	19.3	1.5(1.3,1.9)^c^	1694	35.0	1.6(1.4,1.9)^c^
Middle	1107	16.4	0.9(0.7,1.1)	1581	25.7	2.2(1.8,2.7)^c^	1626	37.9	1.8(1.6,2.1) ^c^
Richer	1450	26.8	1.7(1.4,2.0)^c^	1441	30.7	2.8(2.4,3.4)^c^	1494	47.1	2.7(2.3,3.1) ^c^
Richest	1436	55.8	5.9(5.0,7.0)^c^	1189	58.8	9.1(7.6,11.0)^c^	1330	76.4	9.7(8.2,11.4) ^c^
**Education**	**7927**			**7277**			**7881**		
No education	6517	21.3	1	5712	22.4	1	5249	33.9	1
Primary	994	45.5	3.1(2.7,3.5)^c^	1200	39.8	2.3(2.0,2.6)^c^	2266	55.3	2.4(2.2,2.7) ^c^
Secondary and higher	416	71.6	9.3(7.5,11.7)^c^	365	81.4	15.2(11.6,19.9)^c^	366	91.3	20.0(13.9,28.9) ^c^
**Age**	**7928**			**7275**			**7882**		
15-24	2192	27.1	1.2(1.1,1.4)^b^	1902	30.8	1.5(1.3,1.7)^c^	2009	47.1	1.4(1.2,1.6) ^c^
25-34	3493	29.3	1.4(1.2,1.5)^c^	3378	29.6	1.4(1.2,1.6)^c^	3852	42.5	1.2(1.04,1.3)^b^
35+	2243	23.3	1	1995	23.4	1	2021	38.7	1
**Religion**	**7578**			**7018**			**7628**		
Protestant	1227	25.3	1	1402	29.4	1	1761	40.4	1
Orthodox	4035	27.7	1.1(1.0,1.3)	3247	31.9	1.1(0.9,1.3)	3305	47.8	1.3(1.2,1.5)^c^
Moslem	2316	28.5	1.2(1.0,1.4)^a^	2369	22.8	0.7(0.6,0.8)^c^	2562	38.4	0.9(0.8,1.0)
**Marital status**	**7928**			**7277**			**7880**		
Married	7151	26.8	0.9(0.8,1.0)	6743	27.9	0.8(0.7,1.0)^a^	6738	42.4	0.9(0.8,1.1)
others	777	29.1	1	534	32.0	1	1142	44.3	1
**Birth order**	**7929**			**7276**			**7881**		
1	1354	32.1	1.6(1.4,1.9)^c^	1180	34.9	1.8(1.5,2.1)^c^	1393	54.1	2.0(1.8,2.3) ^c^
2-4	3240	29.4	1.4(1.3,1.6)^c^	3016	30.6	1.5(1.3,1.6)^c^	3451	43.3	1.3(1.2,1.5) ^c^
5+	3335	22.5	1	3080	23.2	1	3037	36.8	1
**Autonomy**	**7867**			**7234**			**7800**		
No	2751	26.3	1	5015	26.2	1	3495	39.5	1
Low	3088	24.0	0.9(0.8,1.0)^a^	1512	25.3	1.0(0.8,1.1)	2005	39.8	1.0(0.9,1.1)
High	2028	32.1	1.3(1.2,1.5)^c^	707	49.1	2.7(2.3,3.2)^c^	2300	50.2	1.5(1.4,1.7)^c^
**Wife beating**	**7913**			**7269**			**7839**		
Yes	6815	25.6	1	6156	26.4	1	6014	37.9	1
No	1098	36.2	1.6(1.4,1.9)^c^	1113	38.4	1.7(1.5,2.0)^c^	1825	58.3	2.3(2.1,2.6) ^c^

^a^p-value < 0.05, ^b^p-value < 0.01, ^c^p-value < 0.001.

**Figure 1:**
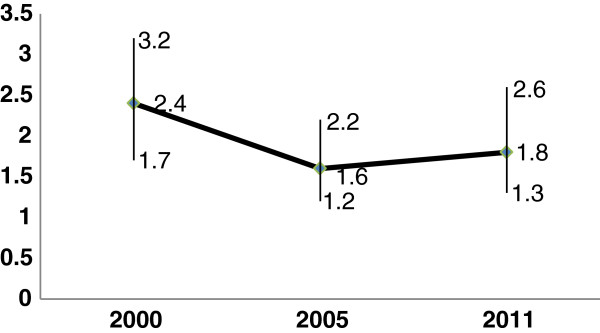
**Adjusted odds ratio and 95% CI of urban**-**rural disparities in ANC unitization in Ethiopia (n = 6744, 2000; n = 6486, 2005 and n = 7611, 2011).** The reference category is rural residence. Adjusted for age, education, economic status, birth order, autonomy and attitude towards wife beatings. Horizontal axis = year and Vertical axis = ORs with 95% CIs.

In all surveys, women from urban areas had more ANC visits than women from rural areas (67.2% vs 21.9% in 2000; 69.7% versus 24.3% in 2005, and 76.5% vs 36.8% in 2011, respectively, Table 2). In the multivariable analysis, in 2000, women from urban areas had 2.4 (95% CI: 1.7, 3.2, p < 0.0001) times the odds to use ANC services compared with women from rural areas, independent of the effects of age, wealth, education, birth order, autonomy and attitude towards wife beatings. Urban–rural disparities in ANC use had lessened in 2005, with an adjusted OR of 1.6 (95% CI: 1.2, 2.2, p = 0.003). There was no further improvement in 2011 (OR: 1.8, 95% CI: 1.3, 2.6; p = 0.001) (Figure 1).

Similarly to the survey undertaken in 2000, in 2005 the lowest rate of ANC use was documented for women from Amhara Regional State (26.7%) and the highest rates were for women from Addis Ababa and Dire Dawa (82.7%). In 2011, the highest rate (90.3%) was documented for women from Addis Ababa and Dire Dawa as well, whereas the lowest rate (30.2%) was found among women from the regions of Afar, Harari and Somali (Table 2). Controlling for age, education, economic status, autonomy and attitude towards wife beatings, the adjusted OR comparing Addis Ababa/Dire Dawa with Amhara were 2.6 (95% CI: 2.0, 3.3, p < 0.0001), 2.1(95% CI: 1.6, 2.8, p < 0.0001), and 3.1(95% CI: 1.9, 5.1; p < 0.0001), in 2000, 2005, and 2011, respectively (Table 3). With the exception of Addis Ababa/Dire Dawa and the Tigray region, inter-regional differences decreased in 2011 compared with previous years; The Oromiya, SNNPR, and the Ben-Gumuz, Gambela regions did better than Amhara in 2000, but differences diminished by 2011. Moreover, the Afar, Harari, and Somali, that had an advantage over Amhara in 2000, did worse in 2005 and 2011 (Table 2).

**Table 3:** Adjusted odds ratios of the effect of region and economic status on antenatal care use at different time points, Ethiopia

**Characteristics**	**2000 (n = 6744)**		**2005 (n = 6486)**		**2011 (n = 7611)**	
	**AOR (95% CI)**	**p-value**	**AOR (95% CI)**	**p-values**	**AOR (95% CI)**	**p-values**
Region*						
Amhara	1	-	1	-	1	-
Tigray	2.5(2.0,3.2)	<0.0001	1.9(1.5,2.4)	<0.0001	2.5(2.0,3.1)	<0.0001
Oromiya	1.4(1.1,1.7)	0.003	0.9(0.7,1.1)	0.252	0.8(0.7,0.9)	<0.0001
SNNPR	1.4(1.1,1.7)	0.004	1.1(0.9,1.3)	0.553	0.9(0.8,1.0)	0.169
Afar, Harari, Somali	1.1(0.9,1.4)	0.390	0.5(0.4, 0.7)	<0.0001	0.6(0.4,0.8)	0.001
Ben-Gumuz, Gambela	1.7(1.3,2.1)	<0.0001	1.2(1.0,1.5)	0.096	1.1(0.7,1.6)	0.684
Addis Ababa, Dire Dawa	2.6(2.0,3.3)	<0.0001	2.1(1.6,2.8)	<0.0001	3.1(1.9,5.1)	<0.0001
Economic status**						
Poorest	1	-	1	-	1	-
Poorer	1.1(0.9,1.4)	0.305	1.5(1.1,1.9)	0.003	1.6(1.3,1.9)	<0.0001
Middle	0.9(0.7,1.1)	0.315	2.1(1.6,2.7)	<0.0001	1.7(1.3,2.1)	<0.0001
Richer	1.5(1.2,1.9)	0.001	2.5(1.9,3.2)	<0.0001	2.2(1.7,2.7)	<0.0001
Richest	2.7(2.1,3.6)	<0.0001	4.4(3.3,6.0)	<0.0001	3.9(2.8,5.5)	<0.0001

*Adjusted for age, education, wealth, autonomy, attitude towards wife beating.

**Adjusted for age, residence, education, birth order, autonomy and attitude towards wife beatings.

A direct association was seen between economic status and ANC visits. In 2000, women from the poorest, poorer and middle economic status households had similar rates of ANC use (range: 16% to 20%), however, rate of ANC use among women from the richest households was dramatically higher at 55.8%. In 2011, women of the poorest households had improved rates of ANC visits at 25.1%, however, women of the richest households had even better improvements with a rate of 76.4% (Table 2).

In the multivariable analysis, economic status remained an independent predictor of ANC use. Controlling for age, residence, education, birth order, autonomy and attitude towards wife beatings, the OR of ANC use among women from households of the highest wealth quintile, compared with the lowest one, were 2.7 (95% CI: 2.1,3.6, p < 0.0001), 4.4 (95% CI: 3.3, 6.0, p < 0.0001), and 3.9 (95% CI: 2.8, 5.5; p < 0.0001) in 2000, 2005, and 2011, respectively (Table 3).

**Figure 2:**
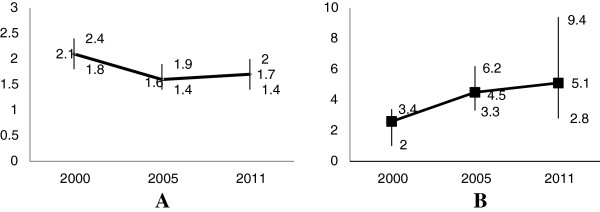
**Adjusted ORs and 95% CI of educational disparities in the use of ANC in Ethiopia (n = 6744, 2000; n = 6486, 2005 and n = 7611, 2011).** Adjusted for age, residence, economic status, birth order, autonomy and attitude towards wife beatings. **A** = primary education versus no education. **B** = secondary or higher education relative to no education. Horizontal axis = year and Vertical axis = ORs with 95% CIs.

The use of ANC had monotonically increased with the level of education of women in all of the three surveys. For instance, in 2005, the rate of ANC use among women with secondary or higher education was 81.4% compared with only 22.4% among women with no education. In 2011, 91.3% of women with secondary or higher education had ANC visits compared to 33.9% of uneducated women (Table 2). Adjusting for age, residence, economic status, birth order, autonomy and attitude towards wife beatings, the OR of ANC use among women with secondary or higher education compared to uneducated women were 1.6 (95% CI: 1.3, 2.0, p < 0.0001), 4.5 (95% CI: 3.3, 6.2; p < 0.0001), and 5.1(95% CI: 2.8, 9.4; p < 0.0001), in 2000, 2005, and 2011, respectively (Figure 2).

Women of no autonomy and low autonomy had similar rates of ANC visits (around 26% in 2005), whereas, women of high autonomy had higher rates (49.1% in 2005) of ANC visits (Table 2). After adjusting for age, residence, wealth, birth order, education and attitude towards wife beatings; there was no association between autonomy and ANC use in 2000 with an OR of 1.1 (95% CI: 0.9, 1.4, p = 0.255) for high autonomy compared with low autonomy. However, the adjusted ORs of ANC use for high autonomy relative to low autonomy were 1.6 (95% CI: 1.3, 2.1, p < 0.0001), and 1.3 (95% CI: 1.1, 1.6, p = 0.003) in 2005 and 2011, respectively.

In accordance, a negative attitude towards wife beating was related to increased ANC use (Table 2). The strength of association between such a negative attitude and ANC attendance had increased from 2000 to 2011 (Table 2). Controlling for age, residence, wealth, birth order, education and autonomy, no association was found between wife beatings and ANC use in 2000 and 2005, yielding OR of 1.1 (95% CI: 0.9, 1.3, p = 0.491) and 1.2 (95% CI: 0.9, 1.4, p = 0.155), respectively. But, in 2011, women with a negative attitude towards wife beatings had an OR of 1.5 (95% CI: 1.2, 1.7, p < 0.0001) for ANC use relative to women with a positive attitude.

### Time trend

To study secular trends, we conducted a multivariable logistic regression where data from all surveys were pooled together, and survey year was treated as a covariate. Controlling for age, residence, economic status, education, attitudes towards wife beatings and economic autonomy, an overall trend of improvement in ANC visits was seen during the study period, with adjusted ORs of 1.2 (95% CI: 1.04, 1.3; p = 0.006) and 2.0 (95% CI: 1.8, 2.2; p < 0.0001) for the years 2005 and 2011 compared to 2000, respectively.

## Discussion

In this nationally-representative study, use of ANC services in Ethiopia has substantially increased since 2000. However, there is evidence of widening gaps by levels of education and economic status. Furthermore, the gap between Addis Ababa/Dire Dawa regions and Amhara region has increased during the study period, whereas gaps between Amhara region and most other regions have almost disappeared. Differences by urbanity remained wide with no clear trend.

Our finding of a gap in ANC use based on economic status is consistent with a study from Nigeria, which measured economic status by household assets and found that women from the very rich households were 6 times more likely to use ANC compared with women from the very poor households [20]. Similarly, a study from India has shown women from the highest economic status households to have a 7-fold increase in ANC visits compared with their counterparts from the lowest economic status households [21]. However, in Namibia the rate ratio of the rich to the poor was 1.06 [22].

In our study, in the year 2011, women with secondary or higher education had five times the odds of having ANC visits compared with women with no education, this is similar with findings from the Madhya Pradesh State of India, where the rates of having at least one ANC check-up of women with higher education (more than secondary) were about 5 times higher than among women with no education [23]. The variations across the levels of education could be due to the capacity of education to increase knowledge of health as well as cognitive ability, to locate needed services and demand quality health care once services are accessed, thus improving the use of health care services [24]. Education may also lead to increased autonomy and decision-making by women [25]. Nonetheless, economic status and work autonomy were controlled for in this analysis of the effect of education and therefore are unlikely to explain the whole association between education and the use of ANC.

Compared to rural women, women from urban areas had OR of 1.8-2.4 for ANC use, adjusted, among others, to wealth and education. Our findings of urban–rural disparities in ANC use are consistent with a study from Ecuador, where Ecuadorian women living in rural areas had an OR of 1.4 (95% CI: 1.0-2.0) for inadequate prenatal care compared with their urban counterparts [26]. However, a study from India reported an absence of independent association between places of residence and ANC use. In India, no urban–rural difference in ANC use was documented in some states of India such as Kerala, Tamil Nadu, Andhra Pradesh and Karnataka. This lack of association was attributed to the assignment of multipurpose health workers in rural areas [27].

The low rate of ANC use among rural women could be attributed to the uneven access to health care between urban and rural areas. As Ethiopia implements strategies to reduce maternal mortality, services are not adequately stretching to rural areas, partly due to lack of roads infrastructure, making distance a crucial factor that gauges the use of maternal health services. In a 2005 DHS report, two thirds of women aged 15–49 who have encountered problems in accessing healthcare cited concern having to take transport, distance to facility, no available provider, financial constraints and concern of the lack of female providers as the major barriers to accessing maternal health care [14]. The cost of the maternal health services could produce the financial constraints. According to Pearson and colleagues, 65% of the health centers they have surveyed in Ethiopia require an out-of-pocket payment for some aspect of care [28], which might be deterring the poor pregnant women from seeking care. Finally, health-seeking behavior of rural and poor women- which were not explored in this study- may have contributed to the urban/rural, economic and regional inequities.

The urban–rural divide had narrowed between 2000 and 2005. However, it was sustained afterwards. This could possibly be due to the initial dramatic expansion of health posts –which are located only in rural areas. In contrast to a 5 fold increase in the number of health posts between 2005 and 2009, there was a 38 fold rise in the number of health posts between 1997 and 2004 [29].

A large variation in the implementation of the HSDP and availability of maternal health services across regional states, and the historical concentration of health services around city states such as Addis Ababa and Dire Dawa may have contributed to the persisting differences across regions. The federal government of Ethiopia dictates that at least 4 health centers per 100 000 people are required in each region of the country. However, the number of health centers per 100 000 people is 0.43 in Somali, 1.56 in Afar, 1.64 in SNNPR and 2.2 in Amhara regions [30]. Even though the federal government has provided technical support to regions such as Afar and Somali, there was no specific budgetary support channeled to maternal health services [29]. This could be an additional mechanism for the wide regional variation in ANC use in Ethiopia, particularly between Addis Ababa/ Dire Dawa and other regions except Tigray.

Our study’s limitations include the inability to assess disparities in the use of 4 ANC visits –which is the new model recommended by World Health Organization [31], lack of assessment of cultural aspects such as acceptability, and attitudes of women and their partners towards ANC. Cultural preference might be a confounding factor, or otherwise could be an interacting variable, in the association between region, residence or economic status and ANC use. Women from different cultural backgrounds may have different beliefs and preferences regarding modern and traditional antenatal care. Lastly, quality of services, previous pregnancy outcomes and problems with on-going pregnancy which could potentially affect the use of ANC were not controlled for in our study.

Our study’s strengths include the nationally representative sample of women of reproductive age. Secondly, we have included community (region, religion and residence), individual (education, birth order and age) and household level (autonomy, economic status and marital status) determinants. In addition, we have controlled for both direct and indirect measures of autonomy, notably, wife beatings as one dimension and work status as a measure of another dimension of autonomy. These constructs are indicators of women’s position in the society as responsible members and their ability to spend their time for their own self-interest [32].

## Conclusions

With regards to policy, the federal government should make effort to put more resources in rural areas and disadvantaged regions such as Somali and Afar. This should be compounded by maneuvers for reducing barriers to access -such as providing economic incentives for poor women or working on the quality of the services. The need for education of girls and women in Ethiopia as a strategy for improving ANC use cannot be overstated. Our study also highlights the need for further studies to explore additional factors which contribute to urban–rural and inter-regional disparities in the use of ANC services. We also recommend additional studies on the mechanism for the initial –between 2000 and 2005- narrowing of urban–rural disparities followed by a sustained gap afterwards, and the disparities in the use of 4 ANC visits in Ethiopia.

## Abbreviations

ANC: Antenatal care; MDG: Millennium development goal; OR: Odds ratio; AOR: Adjusted odds ratio; CI: Confidence interval; DHS: Demographic and health survey; USA: United states of america; WHO: World health organization

## Competing interests

The authors declare that they have no competing interests.

## Authors’ contributions

**YEA** has contributed to the conception of the study. He had participated in the design of the study, analysis of the data and the interpretation of the results. **MRC** was involved in the conception of the study as well as the analysis of the data and the interpretation of the results. Both authors have participated in the preparation of the manuscript. All authors have read and approved the manuscript.

## Authors’ information

**YEA** is currently based at the Department of Health Services Management, Jimma University, Jimma, Ethiopia. His research interest mainly focuses on disparities in health among the different segments of the society that are unjust and unfair. He is also interested in studying the quality of health and medical care as it relates to effectiveness and efficiency. **MRC** is based at the Braun School of Public Health, Hadassah- Hebrew University, Jerusalem, Israel. She trains epidemiology and is interested in the determinants of maternal and neonatal mortality as well as foetal origins of adult disease.

## Pre-publication history

The pre-publication history for this paper can be accessed here:

http://www.biomedcentral.com/1471-2393/13/131/prepub
